# Exploring potential chemical markers by metabolomics method for studying the processing mechanism of traditional Chinese medicine using RPLC-Q-TOF/MS: a case study of Radix Aconiti

**DOI:** 10.1186/1752-153X-7-36

**Published:** 2013-02-22

**Authors:** Yubo Li, Yuming Wang, Lina Su, Lixin Li, Yanjun Zhang

**Affiliations:** 1 Tianjin Key Laboratory of TCM Chemistry and Analysis, School of Traditional Chinese Materia Medica, Tianjin University of TCM, Tianjin 300193, P. R. China; 2 Tianjin Huanhu Hospital, Tianjin 300060, P. R. China

**Keywords:** Metabolomics, Radix Aconiti, Chuan Wu, Reversed phase liquid chromatography/quadrupole time-of-flight tandem mass spectrometry

## Abstract

**Background:**

Pao zhi is a common traditional approach that usually occurs before most herbs are prescribed whereby during processing, secondary plant metabolites are transformed, thus helping to increase potency, reduce toxicity and altering their effects. Using Radix Aconiti (Chuan Wu, CW) as a model herb, suitable chemical markers are crucial for studying the processing mechanisms of these herbs.

**Results:**

In this study, the comprehensive metabolomic characters of CW and Prepared CW (ZCW) by RPLC-Q-TOF/MS were investigated to guarantee clinical safety. Multivariate analyses successfully identified specific metabolite changes between CW and ZCW. In addition, 22 key biomarkers responsible for the detoxifying actions of pao zhi were discovered. The processing mechanism of CW were discussed according to the identified metabolites. This method is efficient, providing more accurate characterisations of traditional pao zhi detoxification.

**Conclusions:**

The proposed strategy proves that RPLC-Q-TOF/MS-based metabolomic analysis does not only explore chemical markers but can also provide a comprehensive understanding of the transformation mechanisms underlying pao zhi.

## Background

Radix Aconiti (Chuan Wu, CW) is the dried mother root of *Aconitum carmichaeli* Debx. This root is an essential drug in Traditional Chinese Medicine (TCM) and has been used for thousands of years. The herb is widely distributed in Sichuan Province (located in southwestern China), and has a wide range of pharmacological effects. Although CW has a limited therapeutic range, it is commonly used to treat various diseases such as collapse, syncope, rheumatic fever, painful joints, gastroenteritis, diarrhoea, oedema, bronchial asthma, and several tumors [1-4]. Prepared CW (ZCW) is traditionally manufactured by boiling raw CW at 100°C for 8 h before drying it. More than 20 commonly used proprietary herbal products from both historical medical literature and modern clinical research reports contain CW or ZCW as main ingredient or auxiliary ingredient. These products include ‘Wutou Tang’, ‘Chuanfu Wan’, ‘Wufu Jiaojiang Tang’, ‘Zhentongning Injection’ and ‘Fengshigutong Jiaonang’ etc.. In TCM, CW and ZCW have different uses and potential toxicity. CW is strongly toxic and is used externally; whereas ZCW has a ‘warning toxicology’, and is taken orally or injected. Therefore, consuming the wrong form of herb may lead to undesirable clinical outcomes. Hence, quality control of this herb is paramount.

Pao zhi is a common approach that usually occurs before most herbs are prescribed whereby during processing. The role of pao zhi is to strengthen the effect, eliminate or reduce the toxicity, facilitate the preparation and storage of drugs. During processing, secondary plant metabolites are transformed, thus helping to increase potency and reduce toxicity, and altering their effects [4]. As a detoxifying measure, Paozhi is necessary to remove the poisonous *Aconitum* alkaloids mainly deriving from the diester diterpene alkaloids (DDAs) including aconitine, mesaconitine and hypaconitine [4]. They can be decomposed into less or non-toxic derivatives through Paozhi that plays an essential role in detoxification. The main mechanisms underlying herb processing were found to be mainly related to changes in composition and/or activity of herb components [5,6]. However, the difference in global metabolomic characters between CW and ZCW remains unclear. This difference restricts further application of ZCW in a clinical environment.

Metabolomics is a branch of science concerned with the total metabolome of integrated biological systems and dynamic responses to alterations of endogenous and/or exogenous factors [7]. The objective of ‘nontargeted’ metabolic profiling analysis is to detect as many metabolites as possible in a certain sample. Several papers have illustrated that metabolomics has been used in evaluating the pharmacological and toxicological effects of aconite products [8,9]. With the development of accurate, precision and new analytical techniques, metabolomics can provide global, comprehensive, detailed and reliable pieces of evidence for further studies and determination on efficacy/toxicity of CWs. Several methods have been developed for analysing aconitine alkaloids in CWs. These methods include high-performance liquid chromatography (HPLC), ultraviolet spectrophotometry (UV) and reversed phase liquid chromatography/quadrupole time-of-flight tandem mass spectrometry (RPLC-Q-TOF/MS) [10-12]. However, research is still limited for content changes of several main alkaloids, while could not exploring potential chemical markers for studying the processing mechanism of CW.

In this study, an approach that uses RPLC-Q-TOF/MS and pattern recognition analysis was developed to rapidly find potential chemical markers for studying the processing mechanism of *Radix Aconiti.* The protocol was executed using three steps. Firstly, this proposed strategy used RPLC-Q-TOF/MS to scan the full metabolic profiling of raw and processed *Radix Aconiti*. Secondly, a multivariate statistical analysis by principal component analysis (PCA) and partial least squares discriminant analysis (PLS-DA) of the mass spectrometry (MS) spectra based on all chemical information was conducted to find potential chemical markers. Lastly, the underlying regulations of pao zhi perturbed metabolic pathways were discussed and the processing mechanism of CW was elucidated according to the results of chemical markers for CW and ZCW. This novel method can be valuable for rapidly exploring potential chemical markers and studying processing mechanisms of herbs.

## Results and discussion

### Acquisition and processing of metabolic profile data

**Figure 1:**
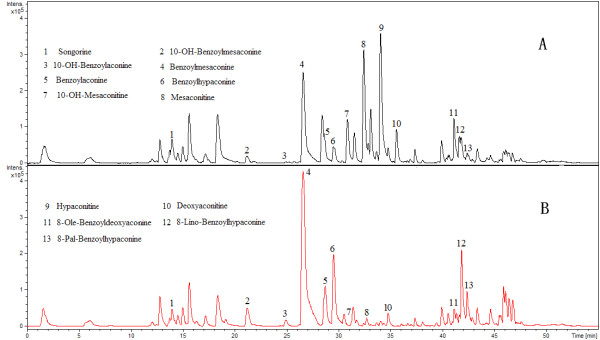
**The representative base peak chromatograms of CW and ZCW by RPLC-Q-TOF/MS in the positive ESI mode. A**, CW; **B**, ZCW.

**Figure 2:**
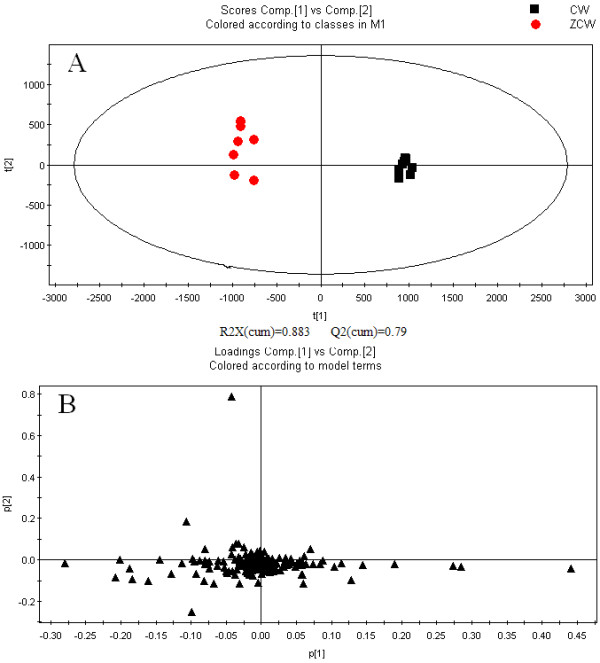
PCA score plots of CW and ZCW (A) and corresponding PLS-DA loading plots (B) of CW and ZCW by RPLC-Q-TOF/MS for pattern recognition.

Global profiling of RPLC-Q-TOF/MS positive ion mode was analysed by RPLC-Q-TOF/MS to compare metabolic difference between CW and ZCW. As shown in Figure 1, low molecular mass metabolites have a good resolution and the baseline can be satisfactorily separated in 60 min. PCA and PLS-DA were used to classify metabolic phenotypes and to identify different metabolites in order to evaluate variation among complex data sets. PCA is an unsupervised pattern recognition method without prior data set information. It retains maximum variance of multidimensional data whilst reducing their dimensionality. PCA score plots were obtained using metabolic data, an obvious separation trend can be observed between CW and ZCW samples in Figure 2A. The loading plot of supervised PLS-DA was used to investigate deeply differences between CW and ZCW and to find potential biomarkers. Figure 2B displays the result of the PLS-DA model using metabolic data. A plot for variable importance parameters (VIPs) was used to identify metabolites according to the order of their contributions to clustering separation [4]. As shown in Table 1, mass spectrometry signals responsible for differentiation were characterised by the values of the VIPs (where a VIP value of >1 is regarded as significant) from the PLS-DA analysis [4]. According to the values of VIPs and the corresponding PLS-DA loading plots, 22 metabolites were identified and selected as potential biomarkers to distinguish striking difference of CW and ZCW.

**Table 1:** I**dentified 22 potential biomarkers between CW and ZCW by RPLC-Q-TOF/MS in positive ESI mode **^**a**^

**No**	**Rt (min)**	**m/z [M + H]**^**+b**^		**Identified compounds**	**Molecular formula**	**MS/MS**	**Structure type**	**Content variance**
		**Measured m/z**	**Theoretical m/z**					
1	30.80	648.3022	648.3015	10-OH-Mesaconitine	C_33_H_45_NO_12_	648[M + H]^+^		↓
						556[M + H–C_3_H_8_O_3_]^+^		
						538[M + H–C_3_H_10_O_4_]^+^		
						528[M + H–C_4_H_8_O_4_]^+^		
						105[M + H–C_26_H_41_NO_11_]^+^		
2	32.36	632.3099	632.3065	Mesaconitine	C_33_H_45_NO_11_	632[M + H]^+^		↓
						572[M + H–C_2_H_4_O_2_]^+^		
						540[M + H–C_3_H_8_O_3_]^+^		
						512[M + H–C_4_H_8_O_4_]^+^		
						105[M + H–C_26_H_41_NO_10_]^+^		
3	32.77	662.3158	662.3171	10-OH-Aconitine	C_34_H_47_NO_12_	662[M + H]^+^		↓
						602[M + H–C_2_H_4_O_2_]^+^		
						570[M + H–C_3_H_8_O_3_]^+^	DDAs	
						542[M + H–C_4_H_8_O_4_]^+^		
						105[M + H–C_27_H_43_NO_11_]^+^		
4	33.97	616.3159	616.3116	Hypaconitine	C_33_H_45_NO_10_	616[M + H]^+^		↓
						584[M + H–CH_4_O]^+^		
						556[M + H–C_2_H_4_O_2_]^+^		
						524[M + H–C_3_H_8_O_3_]^+^		
						105[M + H–C_26_H_41_NO_9_]^+^		
5	35.51	630.3271	630.3273	Deoxyaconitine	C_34_H_47_NO_10_	630[M + H]^+^		↓
						570[M + H–C_2_H_4_O_2_]^+^		
						538[M + H–C_3_H_8_O_3_]^+^		
						510[M + H–C_4_H_8_O_4_]^+^		
						105[M + H–C_27_H_43_NO_9_]^+^		
6	21.1	606.2881	606.2909	10-OH-Benzoylmesaconine	C_31_H_43_NO_11_	606[M + H]^+^		↑
						574[M + H–CH_4_O]^+^		
						556[M + H–CH_6_O_2_]^+^		
						524[M + H–C_2_H_10_O_3_]^+^		
						105[M + H–C_25_H_43_NO_9_]^+^		
7	24.87	620.3020	620.3065	10-OH-Benzoylaconine	C_32_H_45_NO_11_	620[M + H]^+^		↑
						602[M + H–H2O]^+^		
						570[M + H–CH_6_O_2_]^+^		
						538[M + H–C_2_H_10_O_3_]^+^	MDAs	
						105[M + H–C_25_H_45_NO_10_]^+^		
8	26.53	590.2987	590.2960	Benzoylmesaconine	C_31_H_43_NO_10_	590[M + H]^+^		↑
						572[M + H–H_2_O]^+^		
						558[M + H–CH_4_O]^+^		
						540[M + H–CH_6_O_2_]^+^		
						105[M + H–C_24_H_39_NO_9_]^+^		
9	28.65	604.3098	604.3116	Benzoylaconine	C_32_H_45_NO_10_	604[M + H]^+^		↑
						586[M + H–H_2_O]^+^		
						572[M + H–CH_4_O]^+^		
						554[M + H–CH_6_O_2_]^+^		
						105[M + H–C_25_H_41_NO_9_]^+^		
10	29.46	574.3009	574.3011	Benzoylhypaconine	C_31_H_43_NO_9_	574[M + H]^+^		↑
						542[M + H–CH_4_O]^+^		
						510[M + H–C_2_H_8_O_2_]^+^		
						105[M + H–C_24_H_39_NO_8_]^+^		
11	34.58	602.3297	602.2960	Deacetoxy 10-OH-Aconitine	C_32_H_43_NO_10_	602[M + H]^+^		↓
						584[M + H–H_2_O]^+^		
						570[M + H–CH_4_O]^+^		
						552[M + H–CH_6_O_2_]^+^		
12	28.38	572.2858	572.2854	Dehydrated Benzoylmesaconine	C_31_H_41_NO_9_	572[M + H]^+^		↓
						554[M + H–H_2_O]^+^		
						540[M + H–CH_4_O]^+^		
						522[M + H–CH_6_O_2_]^+^		
13	29.56	586.3025	586.3011	Dehydrated Benzoylaconine	C_32_H_43_NO_9_	586[M + H]^+^		↓
						554[M + H–CH_4_O]^+^		
						536[M + H–CH_6_O_2_]^+^		
14	32.52	570.3037	570.3061	Benzoyldeoxyaconine	C_32_H_43_NO_8_	570[M + H]^+^		↓
						552[M + H–H_2_O]^+^		
						520[M + H–CH_6_O_2_]^+^		
15	40.58	850.5086	850.5100	8-Linolen-Benzoylmesaconine	C_49_H_71_NO_11_	850[M + H]^+^		↓
						572[M + H–C_18_H_30_O_2_]^+^		
16	41.05	852.5278	852.5620	8-Ole-Benzoyldeoxyaconine	C_50_H_77_NO_10_	852[M + H]^+^		↓
						570[M + H–C_18_H_34_O_2_]^+^		
17	41.77	836.5316	836.5307	8-Lino-Benzoylhypaconine	C_49_H_73_NO_10_	836[M + H]^+^		↑
						556[M + H–C_18_H_32_O_2_]^+^	LOAs	
18	42.31	812.5295	812.5307	8-Pal-Benzoylhypaconine	C_47_H_73_NO_10_	812[M + H]^+^		↑
						556[M + H–C_16_H_32_O_2_]^+^		
19	42.45	838.5429	838.5464	8-Ole-Benzoylhypaconine	C_49_H_75_NO_10_	838[M + H]^+^		↑
						556[M + H–C_18_H_34_O_2_]^+^		
20	42.81	826.5433	826.5464	8-Pal-Benzoyldeoxyaconine	C_48_H_75_NO_10_	826[M + H]^+^		↑
						570[M + H–C_16_H_32_O_2_]^+^		
21	13.86	358.2358	358.2377	Songorine	C_22_H_31_NO_3_	358[M + H]^+^		↓
						340[M + H–H_2_O]^+^	NEAs	
22	21.76	464.2977	464.3007	14-Acetyl-Talatisamine	C_26_H_41_NO_6_	464[M + H]^+^		↓
						446[M + H–H_2_O]^+^		

^a^ Note: ↑, content increased; ↓, content decreased. DDAs, diester diterpene alkaloids; MDAs, monoester diterpene alkaloids; NEAs, nonester alkaloids; LOAs, lipo-alkaloids. ^b^ Note: all theoretical m/z were calculated by Compass IsotopePattern of Bruke.

### Biomarker characterisation

**Figure 3:**
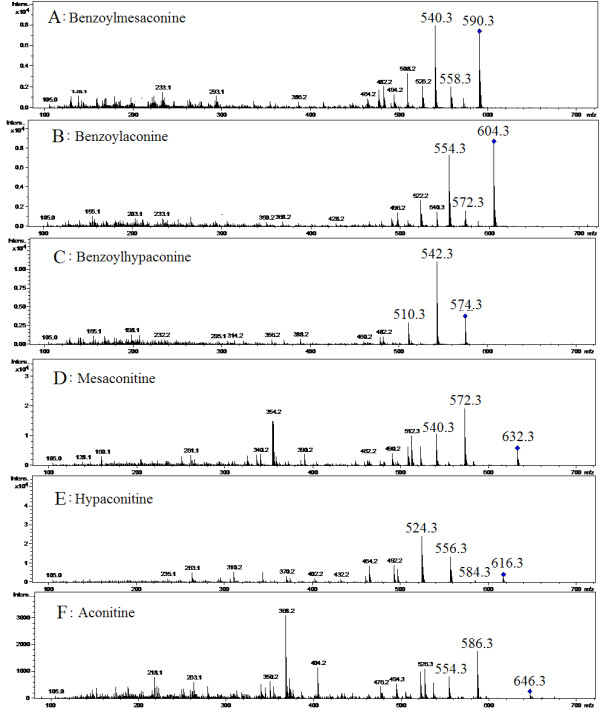
**The MS**^**2 **^**spectra of six standard compounds.** (**A**, Benzoylmesaconine; **B**, Benzoylaconine; **C**, Benzoylhypaconine; **D**, Mesaconitine; **E**, Hypaconitine; **F**, Aconitine).

PCA results displayed as score plots allowed us to compare metabolite profiles of two different groups. PLS-DA loading plots analysis showed distinct metabolites clustered according to the characteristic change of raw or processed sample profiles [4,8,9]. Metabolite identifications were achieved by comparing retention time and MS data (accurate mass, isotopic distribution and fragmentation patterns in positive ion modes) of compounds with alkaloid compounds reported in literature and found in public online databases, or confirmed with standard compounds available in-house (Figure 3). Values for VIPs reflect the influence of each metabolite ion on classification. In addition, values for VIPs are calculated by the formula described in the user’s guide of SIMCA-P. Variables with a VIP value >1 have an above average influence on Y matrix explanation (classification). Therefore, metabolite ions with a VIP value >1 were set aside for further study. Twenty-two ions were identified in the positive mode. Then, the metabolite identification process was illustrated. We chose benzoylmesaconine (an ion at m/z 590.3) as an example to illustrate the biomarker identification process. MS/MS information of 572 [M + H-H_2_O]^+^, 558 [M + H-CH_4_O]^+^, 540 [M + H-CH_6_O_2_]^+^, 508 [M + H-C_2_H_10_O_3_]^+^, 105 [M + H-C_24_H_39_NO_9_]^+^. The molecular formula of the benzoylmesaconine compound was determined as C_31_H_43_NO_10_. As mentioned above, 22 potential biomarkers between CW and ZCW were identified (Table 1). These potential biomarkers are: Dehydrated Benzoylmesaconine, Dehydrated Benzoylaconine, Benzoylaconine, Deoxyaconitine, 10-OH-Benzoylaconine, Hypaconitine, 10-OH-Aconitine, 10-OH-Benzoylmesaconine, Benzoyldeoxyaconine, Benzoylmesaconine, Deacetoxy 10-OH-Aconitine, 10-OH-Mesaconitine, Mesaconitine, Benzoylhypaconine, 8-Pal-Benzoylhypaconine, 8-Linolen-Benzoylmesaconine, 8-Lino-Benzoylhypaconine, 14-Acetyl-Talatisamine, 8-Pal-Benzoyldeoxyaconine, 8-Ole-Benzoylhypaconine, Songorine and 8-Ole-Benzoyldeoxyaconine.

### The processing mechanism of CW

**Figure 4:**
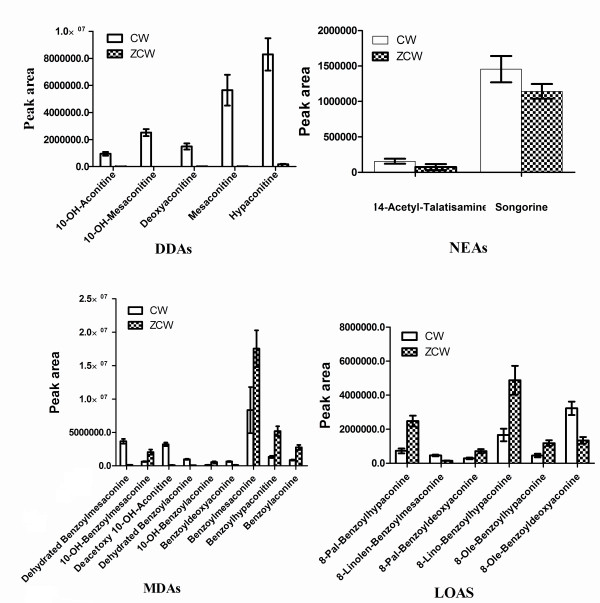
Graphical representation of 22 potential markers between CW and ZCW.

As we know, CW contains poisonous diester diterpene alkaloids (DDAs), less toxic monoester diterpene alkaloids (MDAs) and amine diterpenoid alkaloids (ADAs) [2,4]. When CW is prepared to become ZCW, the main components of DDA and MDA in CW was changed [13,14]. However, the specific change process and metabolite conversion mechanism are still unknown. Thus, we analysed change in metabolic markers to better understand the mechanism of attenuated toxicity. This understanding helps us more clearly explain the pao zhi process. Figure 4 shows that compared with CW, ZCW has lower DDAs concentrations (i.e., Mesaconitine, Deoxyaconitine and Hypaconitine), lower NEAs concentrations (i.e., 14-Acetyl-Talatisamine and Songorine), higher MDAs concentrations (i.e., 10-OH-Benzoylmesaconine, Benzoylmesaconine and Benzoylhypaconitine) and higher Lipo-Alkaloids concentrations (i.e., 8-Pal-Benzoylhypaconine, 8-Lino-Benzoylhypaconine and 8-Pal-Benzoyldeoxyaconine). Dehydrated Benzoylmesaconine, Dehydrated Benzoylaconine and Deacetoxy 10-OH-Aconitine are belonging to the intermediate product of DDAs converted into MDAs(IPDDAs). IPDDA content is lower in ZCW than in CW. The reason may be that the intermediate product is unstable and DDA content in ZCW is lower than that in CW. The detoxification of CW is bound to a hydrolysis procedure. Samples of ZCW are thoroughly boiled with water or steamed for few hours. These results show that pao zhi can play a key role in detoxification. Information about the basic toxicity mechanism of CW and ZCW has also been provided. At present, metabolomics provides more useful information on the basic efficacy/toxicity mechanism of CW and its ZCW, as well as on potential metabolic biomarkers which can be used for the investigation of chemical transformation mechanisms underlying pao zhi.

## Experimental

### Chemicals, reference compounds and samples

Acetonitrile (ACN, HPLC-MS grade) from Merck (Darmstadt, Germany), formic acid (HPLC grade) from Sigma-Aldrich (Steinheim, Germany) and sodium formate from Sigma-Aldrich (St. Louis, MO, USA) were purchased. Ultra-pure water was prepared using a Milli-Q SP system (Millipore, Bedford, MA, USA). Other solvents and chemicals were of analytical grade. CWs were collected from Jiangyou in Sichuan Province, which is the indigenous cultivating region for CW. The identity of all CW samples (root and rhizome) was authenticated to be dried using morphological and histological methods by Dr. Lu Zhang. Preparation of processed CW was carried out according to Chinese Pharmacopoeia (CP) (2010). Voucher specimens of *Aconitum carmichaeli* Debx. and samples used in this study were deposited at Tianjin University of Traditional Chinese Medicine. Six reference compounds were purchased from the National Institute for the Control of Pharmaceutical and Biological Products, China.

### Liquid chromatography

Liquid chromatography was performed with an Agilent 1200 system (Agilent Corp., MA, USA), equipped with a binary solvent delivery system and an autosampler. A mobile phase consisting of water (A) and acetonitrile (B) (each containing 0.1% formic acid) was used. In addition, separation was performed on an RP-C18 column (Agilent Zorbax SB-Aq, 2.1 mm × 100 mm, 1.8 μm particle size). RPLC elution condition was optimised as follows: 2% to 6% B (0 min to 5 min), 6% to 13% B (5 min to 10 min), 13% to 15% B (10 min to 15 min), 15% to 20% B (15 min to 20 min), 20% to 28% B (20 min to 25 min), 28% to 40% B (25 min to 30 min), 40% to 85% B (30 min to 35 min), 85% B (35 min to 40 min), 85% to 2% B (40 min to 42 min), isocratic at 2% B (42 min to 60 min) and finally, washing and reconditioning of the column. Flow rate was set at 0.2 mL/min. The column and autosampler were maintained at 25°C and 10°C, respectively. The injection volume of reference compounds and samples was 2 μL.

### Mass spectrometry

Mass spectrometry analysis was carried out on a time-of-flight mass spectrometer Micro-TOF-QII (Bruker Daltonik GmbH, Germany) using the following setting of tuning parameters: capillary voltage 4.5 kV, drying temperature 180°C, nitrogen flow rate 6 L/min and pressure 0.8 bar. The external calibration with sodium formate was clustered before individual measurements. Mass spectra were acquired in positive electrospray ionization (ESI) mode in a scan range from 100 m/z to 1000 m/z at a sampling rate of 2 Hz. Reference mass was scanned once every five scans for positive data collection.

### Sample preparation of CW and ZCW

Eight samples of raw *Aconitum carmichaeli* Debx. were collected from Jiangyou, Sichuan Province. ZCW was obtained by boiling raw CW at 100°C for 8 h, and then drying it according to CP (2010). The samples were pulverised, passed through a 0.30 mm sieve and stored in a desiccator.

Powder (1.0 g) was extracted with 70% ethanol (10 mL and 8 mL) by extracting it twice before being filtered and combined. The supernatant diluted to 100 mL with deionised water was then passed through a 0.22 mm-filter. The filtrate was stored at 4°C in a refrigerator before being used for RPLC analysis.

### Data processing and pattern recognition analysis

Raw data acquired from RPLC-Q-TOF/MS were pretreated using DataAnalysis 4.0 software (Bruker Daltonics) to find characteristic compounds with molecular features. Furthermore, mass data were exported to ProfileAnalysis 1.1 software (Bruker), which allowed for peak alignment, background noise subtraction and data reduction. Results provided a table of mass and retention time pairs with associated intensities for all detected peaks [15]. The main parameters were set as follows: retention time range 2 min to 55 min, mass range 100 to 1000, mass window 0.5, retention time window 1 min and signal-to-noise (S/N) ratio threshold 5. Variables that did not exist in 80% of participants in one group were filtered [15]. To correct MS response shift during long analysis duration and different sample enrichment factors, data of each sample were normalised, thus ensuring that each sample was represented by a collection of variables to characterise its metabolic pattern before multivariate data analysis.

Normalised data were further exported to SIMCA-P 11.5 demo version software (Umetrics AB, Sweden) for multivariate data analysis [15]. Both PCA and PLS-DA were applied to investigate the metabolic profiles of the samples. PCA is an unsupervised data analysis technique that allows original data to be reduced to a few principal components whilst retaining features that mostly contribute to the variance [16]. By contrast, PLS-DA, is a supervised extension of PCA that uses class information to maximise separation among observation classes. Close sample clustering indicates their compositional similarity, whereas distant sample clustering suggests their diverse metabolomic compositions [17]. The significance of between-group differences for these metabolites was examined by the student’s t-test using the computer software SPSS 13.0 (SPSS Inc., Chicago, USA). P-values less than 0.05 were selected to indicate statistical significance.

## Conclusions

In this study, the comprehensive metabolomic characters of CW and ZCW by RPLC-Q-TOF/MS were investigated to guarantee clinical safety. Multivariate analyses successfully identified specific metabolite changes between CW and ZCW. In addition, 22 key biomarkers responsible for the detoxifying actions of pao zhi were discovered. The processing mechanism of CW were discussed according to the identified metabolites. This method is efficient, providing more accurate characterisations of traditional pao zhi detoxification.

## Abbreviations

CW: Chuan Wu; TCM: Traditional Chinese medicine; ZCW: Prepared CW; HPLC: High-performance liquid chromatography; UV: Ultraviolet spectrophotometry; RPLC-Q-TOF/MS: Reversed phase liquid chromatography/quadrupole time-of-flight tandem mass spectrometry; PCA: Principal component analysis; PLS-DA: Partial least squares discriminant analysis; MS: Mass spectrometry; CP: Chinese pharmacopoeia; ESI: Electrospray ionization; VIPs: Variable importance parameters; DDAs: Diester diterpene alkaloids; MDAs: Monoester diterpene alkaloids; ADAs: Amine diterpenoid alkaloids.

## Competing interests

The authors declare that they have no competing interests.

## Authors’ contributions

All authors contributed to data analyses and drafting of the manuscript. All authors read and approve the final version.
